# Supplementary material for: Identifying s-wave pairing symmetry in single-layer FeSe from topologically trivial edge states
Source: Nat Commun. 2023 Aug 31;14:5302. doi: 10.1038/s41467-023-40931-5 (PMC10471577; doi:10.1038/s41467-023-40931-5)
Supplement: Supplementary file 1 — Supplementary_Information [file 41467_2023_40931_MOESM1_ESM.pdf]

Supplementary Information:

Identifying *s*-wave pairing symmetry in single-layer FeSe from topologically trivial edge states

Zhongxu Wei[1,2#], Shengshan Qin[3,4,5#], Cui Ding[6,2], Xianxin Wu[7], Jiangping Hu[8,4,5], Yujie Sun[1*], Lili Wang[2*] and Qi-Kun Xue[1,2,6*]

[1]Department of Physics, Southern University of Science and Technology, Shenzhen 518055, China

[2]State Key Laboratory of Low-Dimensional Quantum Physics, Department of Physics, Tsinghua University, Beijing 100084, China

[3]School of Physics, Beijing Institute of Technology, Beijing 100081, China

[4]Kavli Institute of Theoretical Sciences, University of Chinese Academy of Sciences, Beijing 100049, China

[5]CAS Center for Excellence in Topological Quantum Computation, University of Chinese Academy of Sciences, Beijing 100049, China

[6]Beijing Academy of Quantum Information Sciences, Beijing 100193, China

[7]CAS Key Laboratory of Theoretical Physics, Institute of Theoretical Physics, Chinese Academy of Sciences, Beijing 100190, China

[8]Beijing National Research Center for Condensed Matter Physics, and Institute of Physics, Chinese Academy of Sciences, Beijing 100190, China

#These authors contributed equally to this work.

*Email: sunyj@sustech.edu.cn; liliwang@mail.tsinghua.edu.cn; xueqk@sustech.edu.cn

Supplementary Note 1: Fitting of Tunneling Spectra

We try to extract superconducting energy gap by fitting the tunneling spectra with a temperature smeared Bardeen–Cooper–Schrieffer (BCS) density of states. Here, we take the blue spectrum at the bottom of Figure 3b in the main text as an example. Before fitting, the normalization is performed by dividing the raw spectrum by its background (blue solid and red dashed curves in Supplementary Figure 1a). The background is extracted from a quintic polynomial fit to the conductance for |V| > 30 mV. The normalized spectrum, denoted by the blue circles in Supplementary Figure 1b, still has a pair of coherence peaks with slightly different intensities. Such electron-hole asymmetry, which is common in iron-based superconductors, may be caused by the band-edge effect due to the relatively shallow band[1,2]. The normalized spectrum is then fitted by the temperature smeared BCS density of states:

$$\frac{\mathrm{d}I}{\mathrm{d}V} \propto \int_{-\infty}^{+\infty} \mathrm{d}E \int_{0}^{2\pi} \frac{1}{2\pi} \mathrm{d}\theta \frac{\partial f(E+eV)}{\partial V} \left| \mathrm{Re}\left(\frac{E}{\sqrt{E^2-\Delta^2(\theta)}} \right) \right|,$$

where f is the Fermi-Dirac function. Since the angle-resolved photoemission spectroscopy measurement has demonstrated that the superconducting gap of single-layer FeSe is moderately anisotropic and a best fit of the gap as a function of angle includes $\cos 2\theta$ and $\cos 4\theta$ terms[3], we take a gap function of

$$\Delta(\theta) = \Delta_0 \left[1 - p_1 \left[1 - \cos\left[4\left(\theta - \frac{\pi}{4} \right) \right] \right] - p_2 \left[1 - \cos\left[2\left(\theta - \frac{\pi}{4} \right) \right] \right] \right],$$

where p_1 and p_2 are the weight of $\cos 4\theta$ and $\cos 2\theta$ terms, respectively.

Based on the above formula, the normalized data is well fitted, as shown by the red curve in Supplementary Figure 1b. More fitting results of normalized tunneling spectra with different superconducting gap magnitude are shown in Supplementary Figure 1c, and corresponding fitting parameters are summarized in Supplementary Table 1. Under such criteria, we obtain the evolution of the gap magnitude near the (11) and (01) edges (Figures 2a and 3a in the main text), as shown in Supplementary Figures 1d and 1e respectively. It is found that gap magnitude decreases with moving close to the edge.

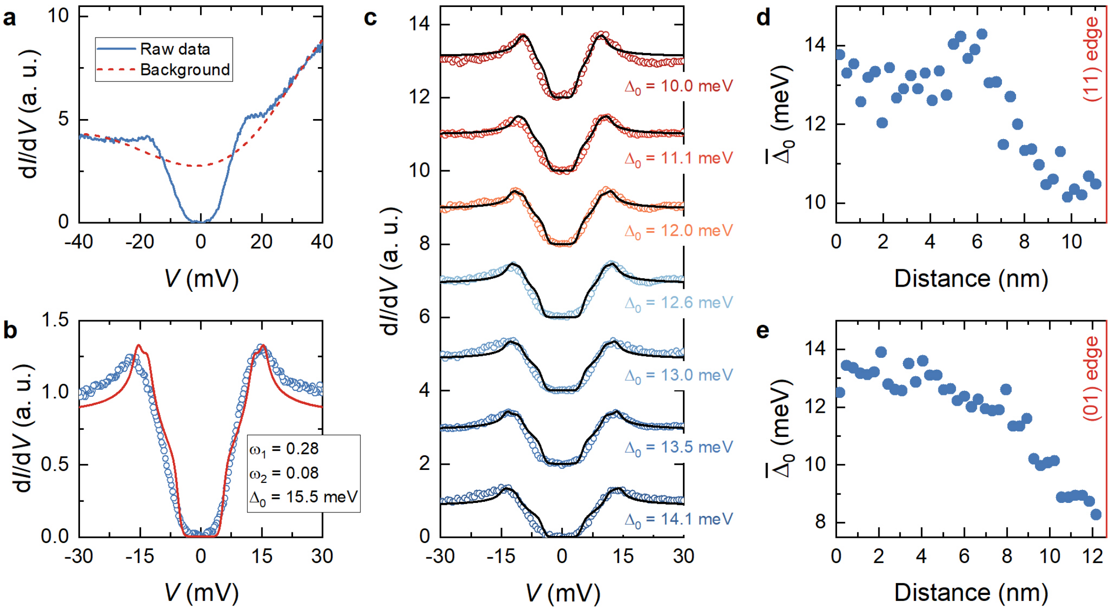

Supplementary Figure 1 | Fitting of tunneling spectra. a, A copy of the blue spectrum at the bottom of Figure 3b in the main text, and its background (red dashed curve). **b**, Normalized data (blue circles) and the fitting result (red curve). **c**, Fitting results of normalized tunneling spectra with various superconducting gap magnitude. **d-e**, Averaged gap magnitude as a function of the distance to the (11) and (01) edges.

Supplementary Table 1 | Fitting parameters of the spectra shown in Supplementary Figure 1c.

No.	Δ (meV)	p_1	p_2
1	10.0	0.28	0.09
2	11.1	0.29	0.09
3	12.0	0.28	0.10
4	12.6	0.25	0.09
5	13.0	0.27	0.09
6	13.5	0.27	0.10
7	14.1	0.29	0.08

Supplementary Note 2: Effects of edge/corner size on the detection of topological modes

The edge/corner modes in the topological state revealed in Ref. [4] are localized states near the edge/corner, and the size of these modes (i.e., the decay length of these modes) are essential for the experimental detections. The decay length of edge modes is mainly determined by the bulk superconducting gap, i.e., Δ_{bulk} in Supplementary Figure 2a, while the decay length of corner modes depends on the superconducting gap at the edges, i.e., Δ_{edge} in Supplementary Figure 2b. More specifically, in such gapped Dirac system, the decay behavior of the localized modes scales as $e^{-\int dr|\Delta(r)/v_F|}$, where $\Delta(r)$ is the position-dependent superconducting gap and v_F is the Fermi velocity. If we roughly ignore the location dependence of the superconducting gap, we have $e^{-\int dr|\Delta(r)/v_F|} \propto e^{-|r/\xi|}$, where $\xi = v_F/\Delta$ is the coherence length.

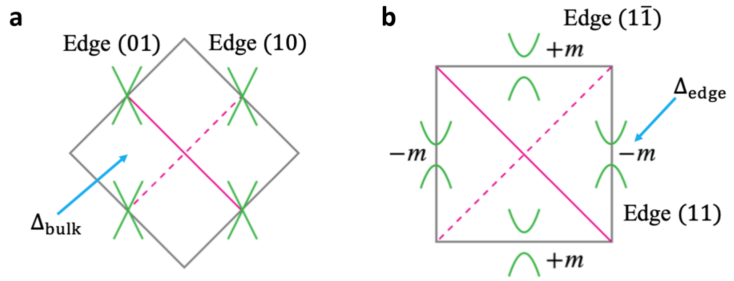

Supplementary Figure 2 | Sketch for the topological edge and corner states in the sign-changing $s_\pm$-wave state. Δ_{bulk} stands for the superconducting gap in the bulk material and Δ_{edge} means the superconducting gap on the (11) and $(1\bar{1})$ edges.

For single-layer FeSe/$SrTiO_3$ system, we have $\Delta_{bulk} \approx 10$ meV and $\xi_{bulk} \approx 3$ nm (Ref. [5]). As for Δ_{edge} and the corresponding coherence length ξ_{edge}, there is no relevant work for reference. Nevertheless, since it has been observed that the superconducting gap at the edge is reduced by less than half compared with that of the bulk [Figures 2c and 3c, Supplementary Figures 1d and 1e], the ξ_{edge} is expected to be larger than ξ_{bulk} but not more than one order of magnitude. Below, we discuss the effects of corner and edge sizes separately:

(1) Edge size——The edges we studied are large enough to stabilize the edge modes. The topographic image of the (01) edge shown in Figure 3a in the main text is taken from the area outlined by the blue box in the topographic image shown in Supplementary Figure 3. It can be seen that the (01) edge we studied extends 31 nm in space and is 17 nm away from the nearest (01) edge. These dimensions are much larger than the coherence length ξ_{bulk}. Therefore, if the single-

layer $FeSe/SrTiO_3$ is in the sign-changing $s_{\pm}$-wave pairing state, our STM measurements can detect clear signals for the Dirac edge modes on the (01) edge.

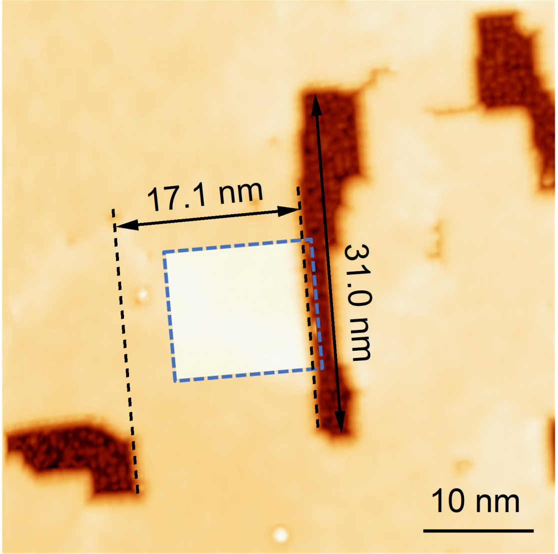

Supplementary Figure 3 | STM topographic images (V_s = 1 V, I_t = 50 pA) of single-layer $FeSe/SrTiO_3$. The (01) edge shown in Figure 3a in the main text is taken from the area outlined by the blue box.

(2) Corner size——Due to the limitations of molecular beam epitaxy, the corner (Figure 4a) we studied with an extension ~ 4 nm may be not large enough to stabilize the corner modes. In this case, the Majorana modes can hybridize with other neighboring topological edge/corner modes, causing the Majorana zero-energy modes to become in-gap bound states distributed symmetrically on both sides of the zero energy[6]. To understand the hybridization, we first consider a circular defect in the sample shown in Supplementary Figure 4a. If the circular defect is large enough, we can apply the mass domain picture described in Ref. [4]. In this case, one would expect four Majorana Kramers' pairs on the circular defect with each pair located at the sign-changing point of the mass term, as indicated by red circles in Supplementary Figure 4a. Then, we simulate the bound states assuming a small circular defect (Supplementary Figures 4b1-4b3) based on the model described in Supplementary Note 5. In the simulation, we take periodic boundary condition in the outer edges. As shown in Supplementary Figures 4c1-4c3, the tunneling spectra collected at the edges of the holes always have in-gap bound states due to the hybridization of corner modes.

Notice that the energy of such in-gap bound states move closer to the superconducting gap edge as the size of the hole becomes smaller.

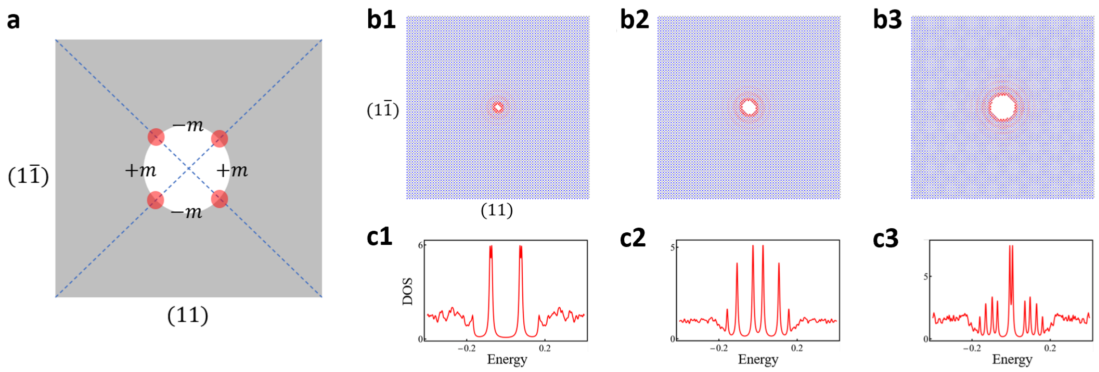

Supplementary Figure 4 | Effect of size on the hybridization of the corner Majorana modes. **a,** Schematic diagram of a circular defect, namely a hole (the white region) embedded in the sample (the gray region). **b1-b3** show the three conditions where the holes have different size, and **c1-c3** show the corresponding local density of states on the edges of the holes. The bulk has a superconducting gap of about 0.2 as shown in Supplementary Figure 9.

If there is only one isolated (11)/($1\bar{1}$) corner connected with a (11)/(10) corner and the (11)/($1\bar{1}$) corner is not large enough, the zero-energy mode at the (11)/($1\bar{1}$) corner will hybridize with the gapless edge mode located at the (01) edge and become a pair of bound states as well.

These in-gap bound states, which can be directly probed by STS, are not observed in Figure 4 and Supplementary Figures 7-8, suggesting that the superconducting states at corners of single-layer FeSe are topologically trivial.

Supplementary Note 3: Additional data for the (01) edge

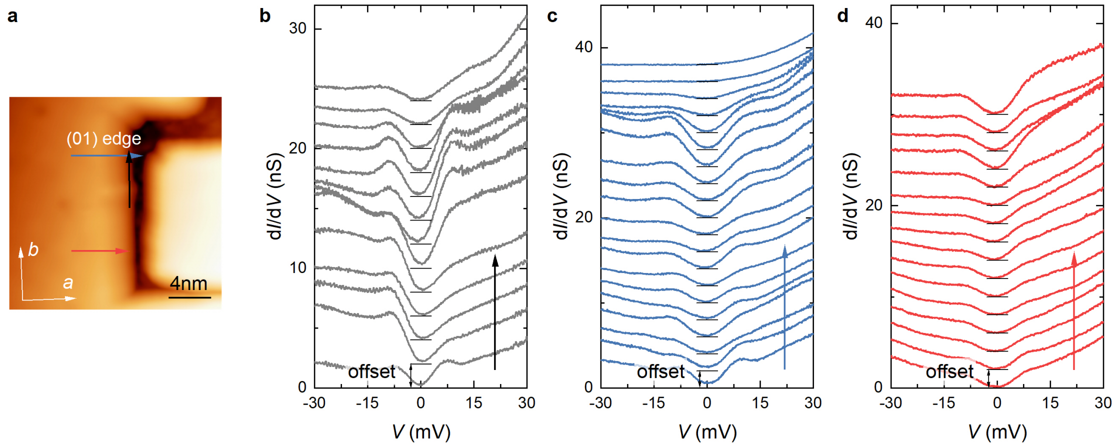

Supplementary Figure 5 | Electronic states at the (01) edge of single-layer FeSe. a, STM topographic image (V_s = 0.5 V, I_t = 100 pA) of the (01) edge. **b-d**, Tunneling spectra (V_s = 50m V, I_t = 500 pA) measured along the black (**b**), blue (**c**) and red (**d**) arrows in **a**. The top two spectra in **c** are taken on the $SrTiO_3$(001) surface. These is no significant change in zero-bias conductance of these spectra.

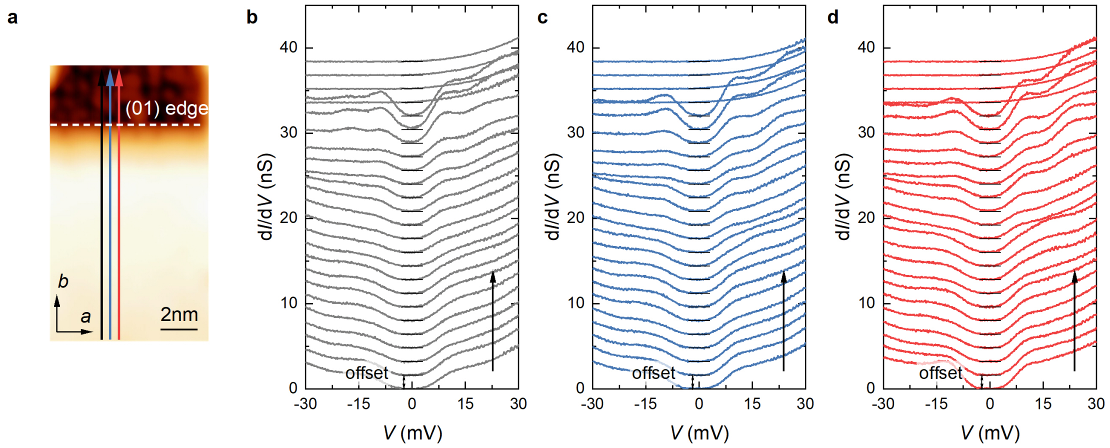

Supplementary Figure 6 | Electronic states at the (01) edge of single-layer FeSe. a, STM topographic image (V_s = 1 V, I_t = 50 pA) of the (01) edge. **b-d**, Tunneling spectra (V_s = 50m V, I_t = 500 pA) measured along the black (**b**), blue (**c**) and red (**d**) arrows in **a**. The top four spectra in **b-d** are taken on the $SrTiO_3$(001) surface. The zero-bias conductance is almost position-independent.

Supplementary Note 4: Additional data for the corner between (11) and $(1\bar{1})$ edges

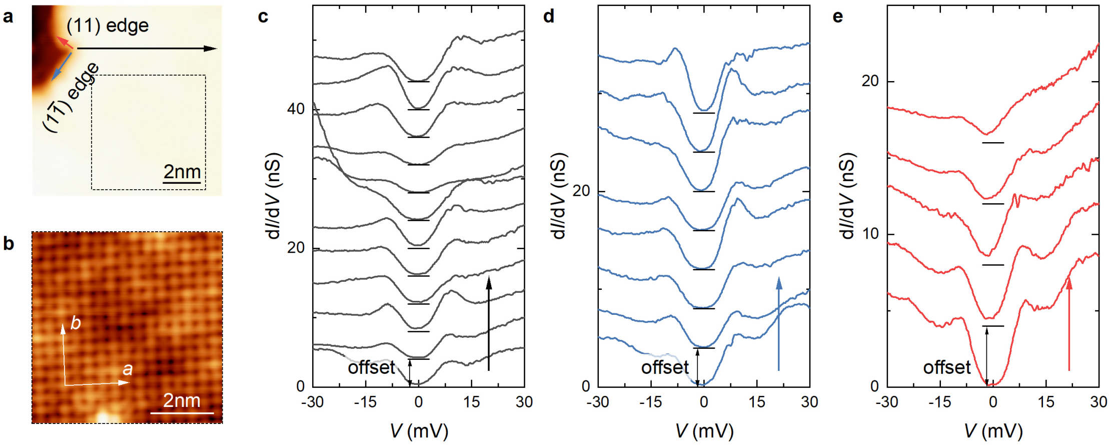

Supplementary Figure 7 | Electronic states at the corner between (11) and $(1\bar{1})$ edges. a, STM topographic image (V_s = 1 V, I_t = 50 pA) of the corner between (11) and $(1\bar{1})$ edges. **b**, Atomically resolved image (V_s = 50 mV, I_t = 500 pA) taken from the area outlined by the black dashed box in **a**. **c**-**e**, Tunneling spectra (V_s = 50 mV, I_t = 500 pA) measured along the black (**c**), blue (**d**) and red (**e**) arrows in **a**. There are no traces of Majorana zero-energy mode and in-gap bound states.

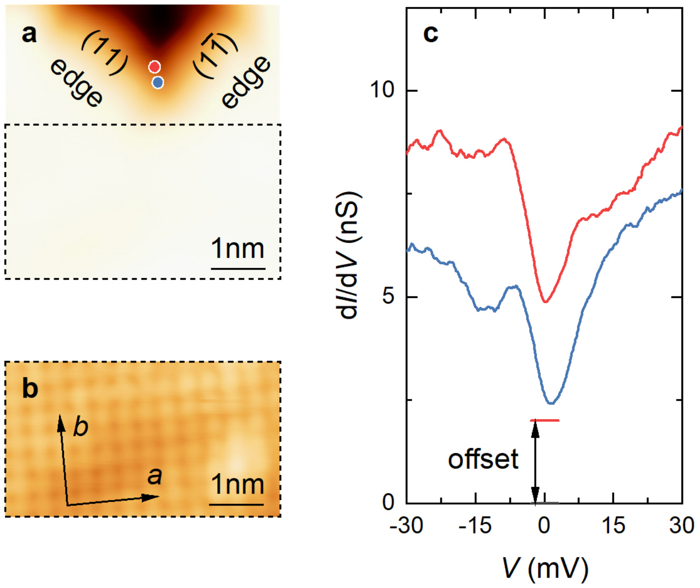

Supplementary Figure 8 | Electronic states at the corner between (11) and $(1\bar{1})$ edges. a, STM topographic image (V_s = 1 V, I_t = 50 pA) of the (11) & $(1\bar{1})$ corner. **b**, Atomically resolved image (V_s = 50 mV, I_t = 500 pA) taken from the area outlined by the black dashed box in **a**. **c**, Tunneling spectra (V_s = 50 mV, I_t = 500 pA) collected at the locations marked by the corresponding colored circles in **a**. The zero-bias conductance peak and in-gap bound states as supposed for $s_{\pm}$-pairing is missing.

Supplementary Note 5: Simulations for the possible influence of the substrate on the experimental observations

We carry out numerical simulations for the possible influence of the STO substrate on the experimental observations of the topological edge/corner modes. In the calculations, instead of using the genuine but complicated model Hamiltonian for the single-layer FeSe which involves all the five *d* orbitals from Fe, we adopt a simple toy model which captures the key topological properties of the sign-changing $s_{\pm}$-wave pairing state. In the toy model, we consider one single *s* orbital at each Fe lattice site and the normal-state Hamiltonian reads as

$$H_0(\mathbf{k}) = [2t(\cos k_x + \cos k_y) - \mu]s_0\sigma_0 - 2R(\sin k_x\, s_2 + \sin k_y\, s_1)\sigma_3 + 4t' \cos\frac{k_x}{2}\cos\frac{k_y}{2} s_0\sigma_1,$$

where s_i and σ_i are the Pauli matrices in the space of the electron spin and two Fe sublattices, respectively. Notice that k_x and k_y are defined according to the primitive lattice translations along the next-nearest-neighbour Fe–Fe directions, i.e., the (10) and (01) directions in the main text. In $H_0(\mathbf{k})$, μ is the chemical potential, t (t') is the hopping between the next-nearest-neighbour (nearest-neighbour) Fe lattice sites, and R is the Rashba-type spin-orbit coupling between the next-nearest-neighbour Fe lattice sites which arises from the mismatch between the Fe lattice sites and the inversion center. $H_0(\mathbf{k})$ respects the symmetry group of the single-layer FeSe and the corresponding band structures are plotted in Supplementary Figure 9a. For the superconducting part, we consider the following *s*-wave pairing

$$H_{pair}(\mathbf{k}) = [\Delta_0 + 2\Delta_1(\cos k_x + \cos k_y)]is_2\sigma_0,$$

where Δ_0 is the onsite pairing and Δ_1 is pairing between the next-nearest-neighbour Fe lattice sites. By tuning the relative magnitude of Δ_0 and Δ_1, we can control the pairing signs on the different Fermi surfaces in $H_0(\mathbf{k})$. As shown in Supplementary Figure 9b, we choose a set of parameters ($\{\Delta_0, \Delta_1\} = \{0.48, 0.2\}$) to simulate the sign-changing $s_{\pm}$-wave pairing state in the iron-based superconductors. For the state in Supplementary Figure 9b, we calculate the superconducting edge modes on the (10) edge and the corner modes between the (11) and $(1\bar{1})$ edges directly and show the results in Supplementary Figures 9c and 9d. Obviously, the above simple model well captures the main features of the second-order topological superconductivity in the sign-changing $s_{\pm}$-wave pairing state in the iron-based superconductors predicted in Ref. [4].

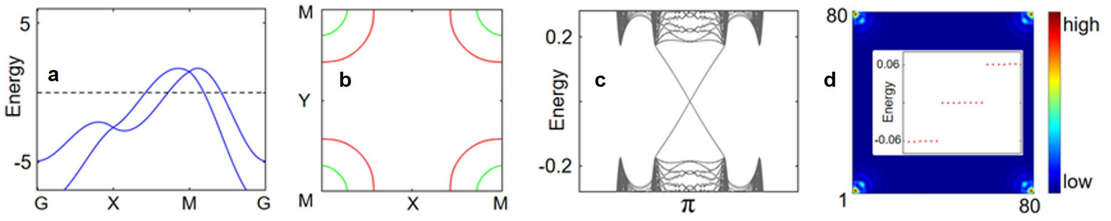

Supplementary Figure 9 | Simulations for the topological edge/corner modes. a shows the band structures calculated from $H_0(\mathbf{k})$ with parameters $\{t, t', R, \mu\} = \{-1.0, 0.4, 0.5, 2.5\}$ with the Fermi energy labeled by the dashed line. **b** shows the Fermi surfaces (colored lines) corresponding to the condition in **a**, and the different colors represent the different pairing signs on the Fermi surfaces corresponding to the superconducting pairing in $H_{pair}(\mathbf{k})$ with $\{\Delta_0, \Delta_1\} = \{0.48, 0.2\}$. The superconducting edge/corner modes corresponding to the condition in **b** are presented in **c**/**d**. **c** shows the edge modes near $k = \pi$ on the (10) edge. Notice that the Dirac edge modes are twofold degenerate. **d** shows the wavefunction profile for the corner Majorana modes with open boundary conditions along the (11) and $(1\bar{1})$ directions, and the inset shows the low-energy spectrum.

In the following, we utilize the above model to simulate the influence of the substrate on the experimental observations, and we adopt the real-space configurations shown in Supplementary Figures 10a-10c in the simulations. The total Hamiltonian for the system reads

$$H_{total}(\mathbf{k}) = H_{sc}(\mathbf{k}) + H_n(\mathbf{k}) + H_{hyb}(\mathbf{k}).$$

Here, $H_{sc} = H_0 + H_{pair}$ is the toy-model superconducting Hamiltonian in the above, $H_n(\mathbf{k}) = 2t_n(\cos k_X + \cos k_Y) + V - \mu$ describes a simple square lattice with t_n being the nearest-neighbour hopping and V being the onsite energy. The hybridization between the superconducting and normal parts is depicted by H_{hyb} containing simply the nearest-neighbour hopping as indicated Supplementary Figure 10c. In modeling the system, we choose proper $V = V_b$ to make the normal region beneath the superconductor metallic since charge transfer is believed to occur between FeSe and STO, and we make the other normal regions insulating ($V = V_n$), i.e. contribute no Fermi surface, as indicated in Supplementary Figures 5-6, Figure 4d in the main text, and Ref. [7]. We present the main numerical results in Supplementary Figure 10. In the calculations, we consider $V_b = 6.0$ and $V_b = 5.0$ in the normal region beneath the superconductor, and in both cases H_n contribute an electron Fermi pocket near the Brillouin zone center. In the latter case, the

normal region has larger electron Fermi pocket which almost compensates the hole Fermi pockets in the superconductor shown in Supplementary Figures 9a and 9b. We also consider the strong ($t_h = 0.5$) and weak ($t_h = 0.2$) hybridization between the superconducting and normal layers. As shown in Supplementary Figure 10, the topological edge/corner modes, especially the corner modes, can be weakened due to its leakage into the substrate; however, finite constant density of states (DOS) are still expected on the (10) edge as indicated in Supplementary Figures 10d1-10d4 and 10e1-10e4. Similarly, a residual zero-bias peak is also expected at the corner between the (11) and $(1\bar{1})$ edges as indicated in Supplementary Figures 10f1-10f4 and 10g1-10g4. Please Note that the higher zero-bias peak in Supplementary Figure 10g4 than in Supplementary Figure 10g3 is due to the finite-size effect (56 × 56 lattice sites for the superconducting layer and 64 × 64 lattice sites for the substrate layer). We have checked the results for larger interlayer coupling ($t_h = 0.8$) and found that the zero-bias peak is much weaker than that in Supplementary Figures 10g3 and 10g4.

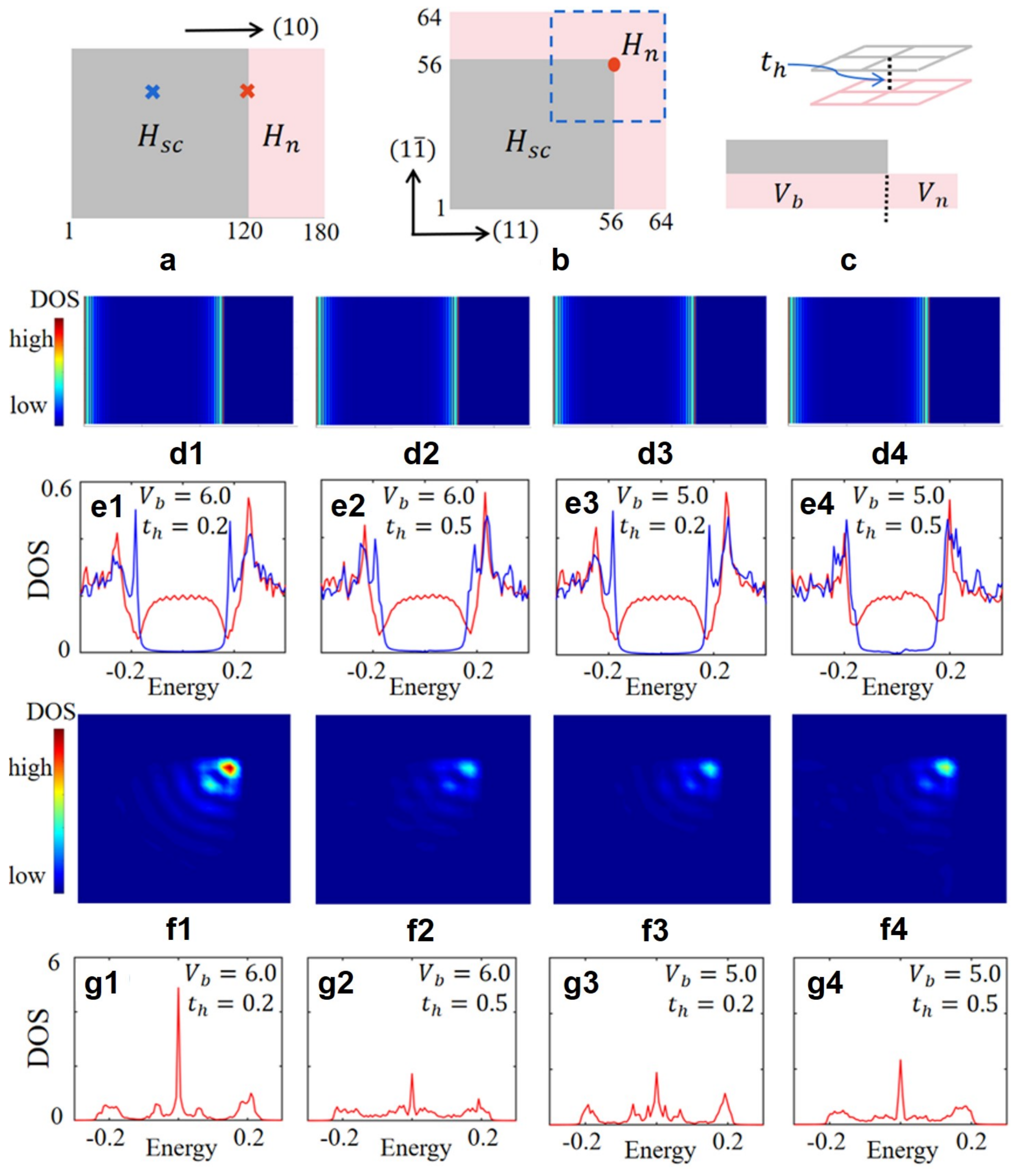

Supplementary Figure 10 | Possible influence of the substrate on the experimental observations of edge/corner modes. **a** and **b** sketch the real-space configurations with which we simulate the influence of the substrate on the edge and corner modes respectively. In **a** we adopt open boundary condition in the (10) direction and periodic boundary condition in the (01) direction, and in **b** we adopt open boundary conditions both in the (11) and $(1\bar{1})$ directions. The gray (pink) part corresponds to the superconducting (normal) region, and the size of the lattice is marked in the figures. **c** shows the side view corresponding to **a** and **b**. Due to the charge transfer between the superconductor and the substrate, the normal region beneath the superconductor can have different onsite energy from the other regions, which is indicated by V_b and V_n in **c**. **d1-d4** show the simulations for the real-space zero-energy DOS of the superconducting edge modes in the configuration in **a**, and the figures share the same color bar. **e1-e4** present the local DOS at the

location marked by × in **a**, and the colored lines in **e1-e4** are in accordance with the locations marked with the corresponding colors. **f1-f4** show the real-space zero-energy DOS of the superconducting corner modes in the region marked by the dashed line in **b**, and the figures share the same color bar. **g1-g4** present the local DOS at the corner marked by the red point in **b**. In the superconducting region described by H_{sc} we adopt the same parameters with these in Supplementary Figure 9, in which case the region has a bulk superconducting gap about 0.2. In the normal region described by H_n, we set $t_n = -1.0$, and set $V_b = 6.0$ ($V_b = 5.0$) for the region beneath the superconductor in **d1-g1** and **d2-g2** (**d3-g3** and **d4-g4**) to make it metallic, and in other regions we set $V_n = 7.5$ to make them insulating. For the hybridization between the superconducting and normal parts described by H_{hyb}, we set $t_h = 0.2$ ($t_h = 0.5$) for the region beneath the superconductor in **d1-g1** and **d3-g3** (**d2-g2** and **d4-g4**).

As a comparison, we also calculate the experimental observations at the edge/corner corresponding to the sign-preserving s-wave pairing state in the presence of the substrate. It is found that the results are completely different from the sign-changing $s_{\pm}$-wave state, as shown in Supplementary Figure 11. Obviously, a U-shape superconducting gap with no in-gap DOS is expected on the (10) edge, and no signature for the zero-bias peak is expected at the corner between the (11) and $(1\bar{1})$ edges.

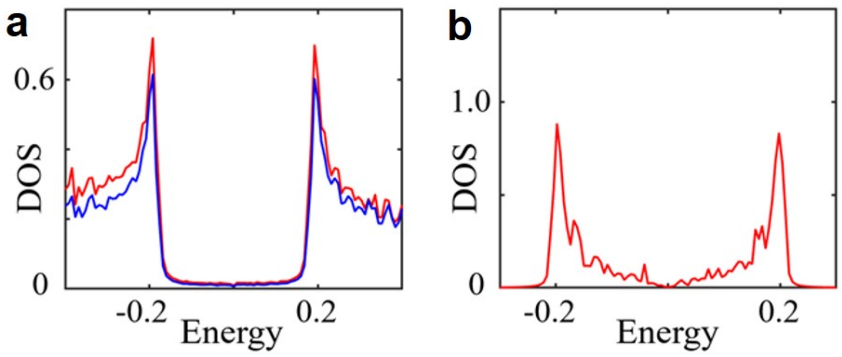

Supplementary Figure 11 | Influence of the $SrTiO_3$ substrate on the experimental observations of edge/corner states. **a** and **b** show the local DOS at the (10) edge and at the corner between the (11) and $(1\bar{1})$ edges in the sign-preserving s-wave pairing state respectively. The colored curves in **a** are calculated at locations marked by the corresponding colored × in Supplementary Figure 10a. The curve in **b** is calculated at the location marked by the red point in

Supplementary Figure 10b. In calculating **a** and **b**, we adopt the superconducting order parameters $\{\Delta_0, \Delta_1\} = \{0.2, 0.0\}$ in $H_{pair}(\mathbf{k})$, and the other parameters are all the same with these in Supplementary Figures 10e4 and 10g4.

Finally, it is worth mentioning that although the model we adopted in the calculations is simpler compared to the actual FeSe/STO system, the results can still provide us important implications as the model captures the main physics near the Fermi energy. Therefore, it is reasonable to conclude that the topological edge/corner modes can serve as experimental observables to distinguish the pairing symmetry of the single-layer FeSe system even in the presence of the substrate.

Supplementary Note 6: Effects of decrease in gap magnitude near the edge on the detection of edge modes

The gradual decrease of the superconducting gap when approaching the edge (Figures 2c and 3c, Supplementary Figures 1d and 1e) implies that the superconducting transition temperature (T_c) at the edge decreases compared with the T_c at the terrace. Here, we demonstrate that the reduction of superconducting gap/T_c will not affect the detection of edge modes from two aspects:

(1) Impact of theoretical applicability——The reduced superconducting gap near the edge can solely modify the topological edge/corner modes quantitatively and it cannot make them disappear, as long as the system is in the sign-changing $s_{\pm}$-wave pairing state. To confirm this, we carry out numerical simulations based on the model described in the Supplementary Note 5 and we take the same parameters with those in Supplementary Figure 9. To simulate the reduced superconducting gap near the edge, we assume the position-dependent behavior of the superconducting orders in the following form

$$\Delta(r) = \tilde{\Delta}\left(0.5 + 0.5 * \tanh\frac{r}{R_0}\right)$$

where r is the distance to the edge, and $\tilde{\Delta}$ is the superconducting order parameter in the bulk ($\tilde{\Delta}$ stands for both the on-site pairing Δ_0 and the next-nearest-neighbor pairing Δ_1). In the above formula, R_0 characterizes the range near the edge where the superconducting order is suppressed. One can check that the superconducting gap near the edge is about half of the superconducting gap in the bulk, which well mimics the experimental results (Figures 2c and 3c, Supplementary Figures 1d and 1e). We present the corresponding numerical results in Supplementary Figure 12. Obviously, the Dirac edge modes at the (10) edge and the Majorana modes at the corner between the (11) and $(1\bar{1})$ edges maintain. The reduced superconducting gap near the edge merely modifies the superconducting energy spectrum of the edge/corner states as shown in Supplementary Figures 12a and 12b, and makes the corner states more extended as presented in Supplementary Figure 12c.

We want to emphasize that the reduced superconducting gap near the edge cannot change the topological property of the system, as long as the superconductor keeps fully gapped. In other words, if the reduced superconducting gap near the edge changes the topology (the topological property of the superconductor far away from the edge and the topological property of the

superconductor near the edge are different, such as it being sign-changing $s_{\pm}$-wave in the bulk and sign-preserving s-wave near the edge), a topological phase transition must occur and the topological edge/corner modes must appear at some distance away from the edge correspondingly. However, in the experimental results presented in the manuscript one can find that, the single-layer FeSe/$SrTiO_3$ system is fully gapped everywhere in the real space, indicating the non-existence of such topological phase transition.

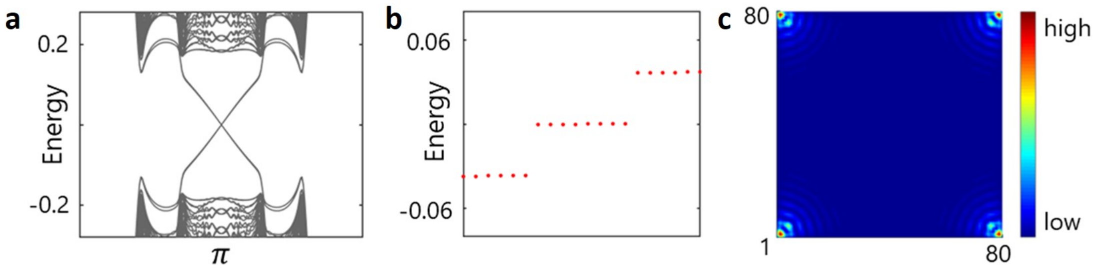

Supplementary Figure 12 | Superconducting edge and corner modes in the case where the suppressed superconducting gap near the edges [$\Delta(r) = \tilde{\Delta}(0.5 + 0.5 * \tanh\frac{r}{R_0})$] is considered. **a** shows the superconducting spectrum when open-boundary condition is considered along the (10) direction. **b** shows low-energy spectrum when open-boundary condition is considered both along the (11) and $(1\bar{1})$ directions. **c** presents the profile of the wave function for the zero-energy modes in **b**.

(2) Impact of data analysis——A direct effect of decrease in T_c at the edge is the increase in the ratio of the experimental temperature T_{exp} to T_c. When T_{exp}/T_c is close to 1, the zero-bias conductance (ZBC) of tunneling spectrum will not be zero due to partial condensation of electrons induced by thermal fluctuations, which makes edge/corner modes difficult to identify. Assuming that the ratio $2\Delta/k_B T_c$ of single-layer FeSe is a constant, a reduction of nearly half of the superconducting gap at the edge (Figures 2c and 3c, Supplementary Figures 1d and 1e) implies that the T_c at the edge becomes half of 65 K, i.e., about 33 K. Here, the critical temperature of 65 K is the gap-close temperature of single-layer FeSe, which has been revealed by STM and ARPES investigations[8-10]. We can then estimate that the T_{exp}/T_c at the edge is about 4.8 K/33 K = 0.15.

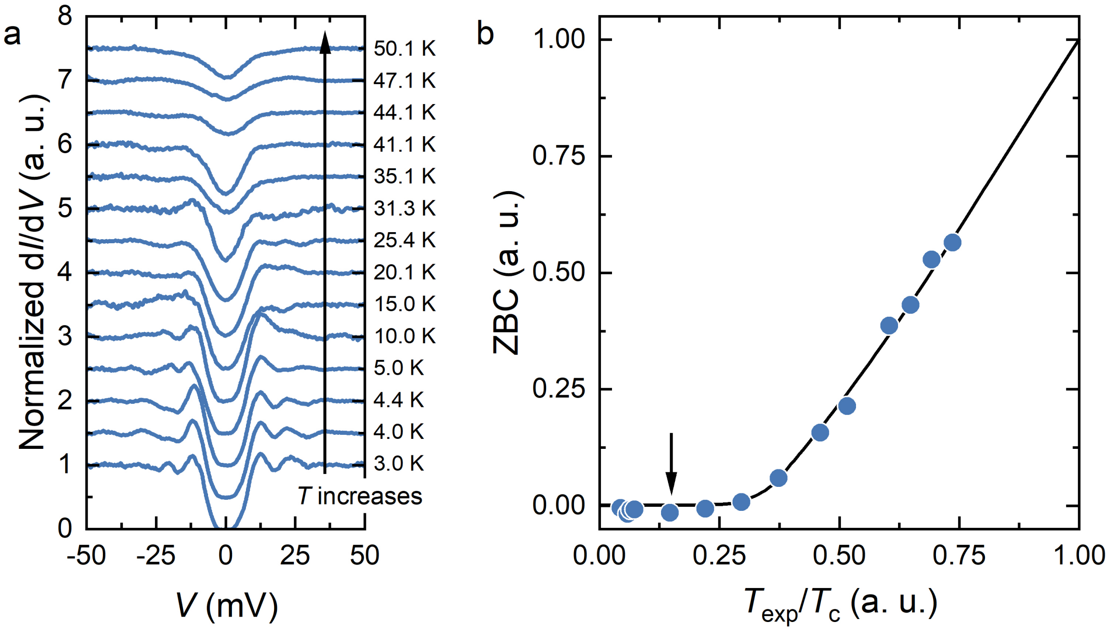

Supplementary Figure 13 | Temperature-dependent tunneling spectra of single-layer FeSe. a, Temperature-dependent tunneling spectra collected on single-layer FeSe. The spectra are shifted along the y axis at a fixed value of 0.5. **b**, Temperature-dependent zero-bias conductance extracted from the tunneling spectrum shown in **a**.

Supplementary Figure 13a shows the temperature-dependent tunneling spectra of single-layer FeSe (see Ref. [9] for experimental details). Supplementary Figure 13b presents the temperature-dependent ZBC extracted from the spectra shown in Supplementary Figure 13a. Obviously, as T_{exp}/T_c increases, the ZBC first remains unchanged ($T_{exp}/T_c < 0.25$), then gradually increases and approaches 1 ($T_{exp}/T_c > 0.25$). As indicated by the black arrow in Supplementary Figure 13b, a T_{exp}/T_c of 0.15 does not lead to a significant change in ZBC. In other words, the reduced T_c at the edge will not have a significant impact on the zero-bias conductance because the reduced T_c is still much larger than the experimental temperature T_{exp}. Therefore, the decrease of T_c at the edge (from 65 K to 33 K) will not affect the detection of edge modes.

Supplementary Note 7: Sensitivity of our tunneling junction to differential conductance anomalies

Anomalies in the superconducting gap and zero-bias conductance are observed in the same region marked by the white boxes in Figures 2c and 2d, which may be caused by local defects (white box in Figure 2a). Supplementary Figure 14a is a reproduction of Figure 2a in the manuscript. The atomically resolved image presented in Supplementary Figure 14a shows bright lobes on adjacent top-layer Se sites, which may be caused by some perturbation at the Fe site[11,12]. Supplementary Figure 14b shows the tunneling spectrum collected at the green across near the local defect. The tunneling spectrum has a conductance peak at -4.0 mV (dashed line), which is similar to the characteristics of Se_{Fe} antisite defect[13]. Therefore, we preliminarily attributed the anomalies on the topographic image to Se_{Fe} antisite defect.

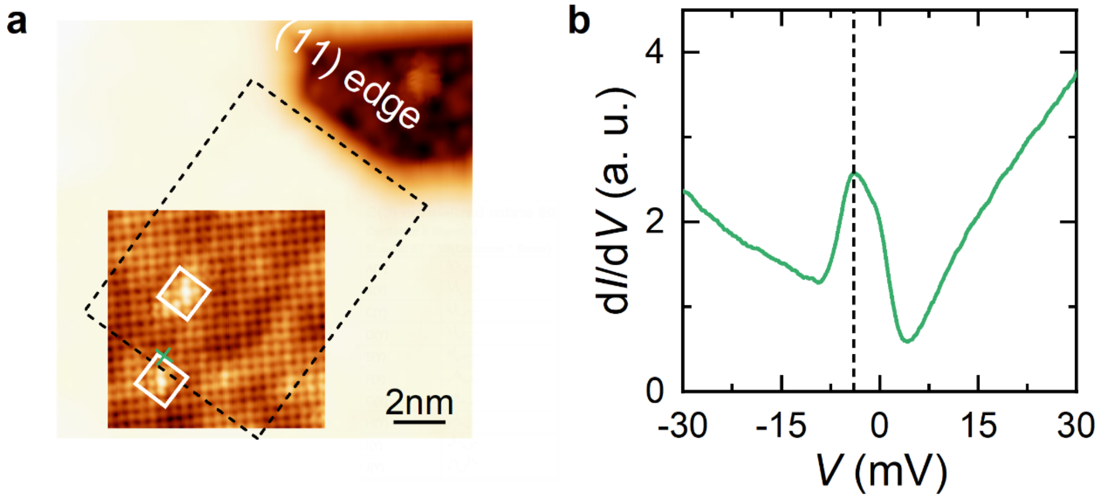

Supplementary Figure 14 | In-gap bound states induced by local defects. a, A copy of Figure 2a in the manuscript. **b**, The tunneling spectrum collected at the green cross shown in **a**.

We would like to emphasize that the exact origin of the local defects is not the focus of this work and is irrelevant to the topological properties studied here. How these defects interact with electrons to generate bound states is beyond the scope of this paper. Nevertheless, the successful capture of anomalies in topographic image and electronic state proves that our equipment (or setup conditions) has sufficient spatial resolution and energy resolution to detect potential edge/corner modes. On this basis, no obvious anomalies in topographic image and superconducting gap are observed near the (01) edge (Figures 3a and 3c), indicating the high quality of the edge and

providing an excellent opportunity to study the edge modes. Therefore, the position-independent zero-bias conductance (Figures 3d and 3e) intrinsically reveals the absence of topologically non-trivial edge modes and provides solid evidence for the sign-preserving *s*-wave pairing symmetry of single-layer FeSe.

Supplementary Note 8: Analysis of spectral weight of the normalized tunneling spectra

Z. Z. Ge et al. argues a nodeless *d*-wave pairing based on the observation of decreased gap magnitude and emergent conductance peak at the (01) edge but not at the (11) edge[14]. The conductance peak is located near the superconducting gap and can only be resolved after a normalization done by subtracting the spectrum far from the edge. In this work, however, we observe the decrease of Δ at both (01) and (11) edges (Figures 2c and 3c in the main text and Supplementary Figures 1d and 1e), which may be due to the lattice discontinuity (Figures 2a and 3a in the main text). In addition, we capture the conductance peak in normalized tunneling spectra at both (01) and (11) edges. Supplementary Figure 15c shows a set of tunneling spectra measured along the black arrow in Supplementary Figure 15a. Using the normalization method proposed by Ref. [14], that is, subtracting each spectrum (#1 ~ #23) from the spectrum far from the edge (#1), a pair of conductance peaks with energy close to Δ are identified near the (11) edge (dashed lines in Supplementary Figure 15d). Similar conductance peak can be obtained near the (01) edge (Supplementary Figures 15b and 15e). Here, we want to point out that such conductance peak is not related to topological properties, but is a natural consequence of the reduction in superconducting gap magnitude, which can be reproduced by a modified BCS model. Supplementary Figure 15f shows two spectra simulated by a temperature smeared BCS density of states with an anisotropic gap function (See Supplementary Note 1 for details). Based on the evolution of gap magnitude near the edges (Supplementary Figures 1d and 1e), Δ_0 = 12 and 10 meV are used to mimic the tunneling spectra away from (red) and near (blue) the edge, respectively. The conductance peak indicated by dashed lines emerge naturally after subtracting the red spectrum from the blue spectrum (Supplementary Figure 15g). Therefore, great care should be taken in determining pairing symmetry using the spectral weight obtained by such normalization method.

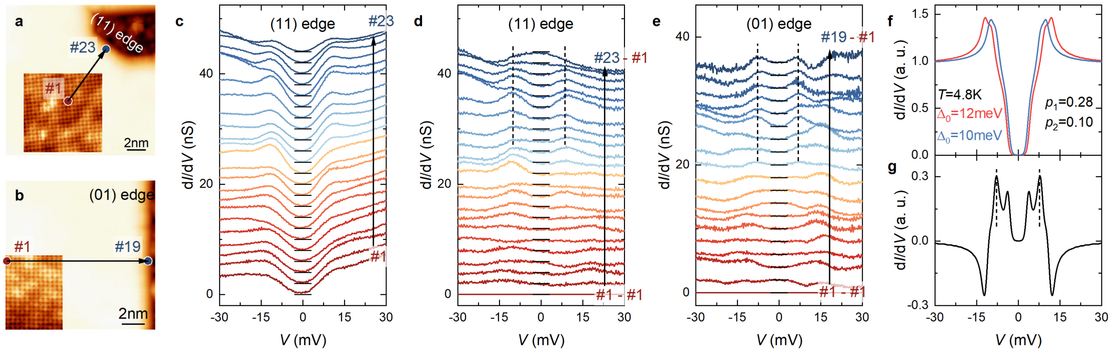

Supplementary Figure 15 | Spectral weight of the normalized tunneling spectra. a-b, STM topographic images (V_s = 1 V, I_t = 50 pA) of the (11) and (01) edges. Insets: atomically resolved images (V_s = 50 mV, I_t = 500 pA). **c-d**, Raw and normalized spectra collected along the black arrow in **a**. **e**, Normalized spectra collected along the arrow in **b**. **f**, Spectra with different gap values simulated by the temperature smeared BCS density of states as described in Supplementary Note 1. The red and blue curves mimic the tunneling spectra away from and near the edge, respectively. **g**, Normalized spectra obtained by subtracting the red spectrum from the blue spectrum in **f**.

Supplementary Note 9: Correlation between edge state and pairing symmetry

We note that a previous work (Ref. [15]) has investigated the topological properties of single-layer FeSe and discovered the 1D topological edge states at the M point of the Brillouin zone. It should be emphasized that our findings do not conflict with those in Ref. [15]. Notice that the topological phase in Ref. [15] is associated with the spin-orbit coupling gap in the antiferromagnetic state, and the topological band lies ~ 100 meV below the Fermi surface, which is well beyond the energy window of the superconducting gap (~ 15 meV). Therefore, the topological edge states revealed in Ref. [15] are not related to the specific properties of superconductivity and cannot reflect the relevant information of superconducting pairing. In contrast, our theoretical[4] and experimental works focus on the superconductivity near the Fermi energy and the possible topological edge/corner states located within the superconducting gap which are closely related to the details of pairing symmetry. According to our theoretical calculations[4], a sign-changing $s_{\pm}$-wave pairing leads to a second-order superconducting state at the (01) edge and at the corner between the (11) and $(1\bar{1})$ edges, while the sign-preserving *s*-wave state remains topologically trivial even in the presence of the inversion symmetric Rashba SOC. The tunneling spectra we collected at edges and corners exhibit full gap and substantial dip near the Fermi energy, respectively, demonstrating the absence of topologically non-trivial edge/corner modes within the superconducting gap, and thus providing solid evidence for the sign-preserving *s*-wave pairing in single-layer FeSe.

Supplementary Reference

1. Wang, Z. *et al.* Close relationship between superconductivity and the bosonic mode in $Ba_{0.6}K_{0.4}Fe_2As_2$ and $Na(Fe_{0.975}Co_{0.025})As$. *Nat. Phys.* **9**, 42-48 (2012).
2. Chen, C. *et al.* Observation of discrete conventional Caroli-de Gennes-Matricon states in the vortex core of single-layer $FeSe/SrTiO_3$. *Phys. Rev. Lett.* **124**, 097001 (2020).
3. Zhang, Y. *et al.* Superconducting gap anisotropy in monolayer FeSe thin film. *Phys. Rev. Lett.* **117**, 117001 (2016).
4. Qin, S., Fang, C., Zhang, F.-C. & Hu, J. Topological superconductivity in an extended *s*-wave superconductor and its implication to iron-based superconductors. *Phys. Rev. X* **12**, 011030 (2022).
5. Fan, Q. *et al.* Plain *s*-wave superconductivity in single-layer FeSe on $SrTiO_3$ probed by scanning tunnelling microscopy. *Nat. Phys.* **11**, 946-952 (2015).
6. Chiu, C.-K., Machida, T., Huang, Y., Hanaguri, T. & Zhang, F.-C. Scalable Majorana vortex modes in iron-based superconductors. *Sci. Adv.* **6**, eaay0443 (2020).
7. He, S. L. *et al.* Phase diagram and electronic indication of high-temperature superconductivity at 65 K in single-layer FeSe films. *Nat. Mater.* **12**, 605-610 (2013).
8. Tan, S. *et al.* Interface-induced superconductivity and strain-dependent spin density waves in $FeSe/SrTiO_3$ thin films. *Nat. Mater.* **12**, 634-640 (2013).
9. Zhang, W. H. *et al.* Interface charge doping effects on superconductivity of single-unit-cell FeSe films on $SrTiO_3$ substrates. *Phys. Rev. B* **89**, 060506(R) (2014).
10. Xu, Y. *et al.* Spectroscopic evidence of superconductivity pairing at 83K in single-layer $FeSe/SrTiO_3$ films. *Nat. Commun.* **12**, 2840 (2021).
11. Choubey, P., Berlijn, T., Kreisel, A., Cao, C. & Hirschfeld, P. J. Visualization of atomic-scale phenomena in superconductors: Application to FeSe. *Phys. Rev. B* **90**, 134520 (2014).
12. Huang, D. *et al.* Dumbbell Defects in FeSe Films: A Scanning Tunneling Microscopy and First-Principles Investigation. *Nano Lett.* **16**, 4224-4229 (2016).
13. Liu, C. *et al.* Extensive impurity-scattering study on the pairing symmetry of monolayer FeSe films on $SrTiO_3$. *Phys. Rev. B* **97**, 024502 (2018).
14. Ge, Z. Z., Yan, C. H., Zhang, H. M., Agterberg, D., Weinert, M. & Li, L. Evidence for *d*-wave superconductivity in single layer $FeSe/SrTiO_3$ probed by quasiparticle scattering off step edges. *Nano Lett.* **19**, 2497-2502 (2019).
15. Wang, Z. F. *et al.* Topological edge states in a high-temperature superconductor $FeSe/SrTiO_3(001)$ film. *Nat. Mater.* **15**, 968-973 (2016).
